# Ultrasmall gold and silver/gold nanoparticles (2 nm) as autofluorescent labels for poly(D,L-lactide-*co*-glycolide) nanoparticles (140 nm)

**DOI:** 10.1007/s10856-020-06449-8

**Published:** 2020-11-28

**Authors:** Karolin Wey, Matthias Epple

**Affiliations:** Inorganic Chemistry and Center for Nanointegration Duisburg-Essen (CeNIDE), University of Duisburg-Essen, Universitätsstr. 5-7, 45117 Essen, Germany

## Abstract

Ultrasmall metallic nanoparticles show an efficient autofluorescence after excitation in the UV region, combined with a low degree of fluorescent bleaching. Thus, they can be used as fluorescent labels for polymer nanoparticles which are frequently used for drug delivery. A versatile water-in-oil-in-water emulsion-evaporation method was developed to load poly(D,L-lactide-*co*-glycolide) (PLGA) nanoparticles with autofluorescent ultrasmall gold and silver/gold nanoparticles (diameter 2 nm). The metallic nanoparticles were prepared by reduction of tetrachloroauric acid with sodium borohydride and colloidally stabilised with 11-mercaptoundecanoic acid. They were characterised by UV–Vis and fluorescence spectroscopy, showing a large Stokes shift of about 370 nm with excitation maxima at 250/270 nm and emission maxima at 620/640 nm for gold and silver/gold nanoparticles, respectively. The labelled PLGA nanoparticles (140 nm) were characterised by dynamic light scattering (DLS), scanning electron microscopy (SEM), and UV–Vis and fluorescence spectroscopy. Their uptake by HeLa cells was followed by confocal laser scanning microscopy. The metallic nanoparticles remained inside the PLGA particle after cellular uptake, demonstrating the efficient encapsulation and the applicability to label the polymer nanoparticle. In terms of fluorescence, the metallic nanoparticles were comparable to fluorescein isothiocyanate (FITC).

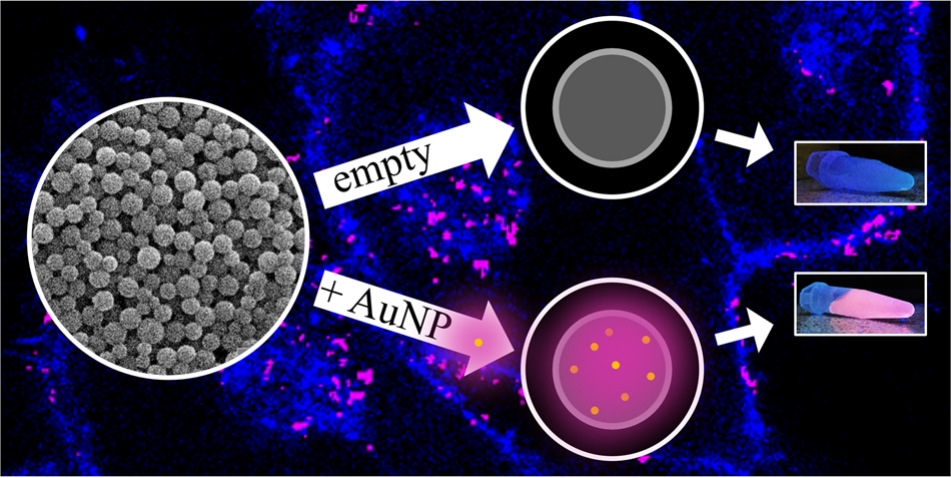

## Introduction

The delivery of functional molecules such as biomolecules or water-insoluble drugs is an ongoing challenge in medicine. Usually, pure biomolecules cannot penetrate the cell membrane and are sensitive to enzymatic degradation [1, 2]. Biodegradable polymeric nanoparticles can be used as carriers for a variety of drugs and molecules [3–5]. After being taken up by a cell, e.g. by endocytosis or phagocytosis, the polymer particle is degraded and the drug is released [3]. Poly(D,L-lactide-*co*-glycolide) (PLGA) is a polymer that is frequently used for intracellular drug delivery [6–9]. It is biodegradable, biocompatible and also approved for human use by the *American Food and Drug Administration* and the *European Medicine Agency* [3, 4, 10, 11]. Therefore, it is one of the most frequently investigated and applied biodegradable polymers in many medical applications including drug delivery, osteosynthesis and tissue engineering [3, 12, 13]. PLGA is hydrolysed inside the body to lactic acid and glycolic acid which are both biodegradable metabolites [3]. To trace the pathway of such drug-loaded nanoparticles inside cells or tissues, optical methods like fluorescence microscopy are usually employed, therefore a fluorescent labelling with suitable fluorophores is necessary [1, 14–18].

In confocal microscopy, only a limited number of dyes is applicable to capture images simultaneously, as they show spectral crossover between the channels [19]. Classic organic fluorescence dyes have a small Stokes shift (the difference between absorption maximum and emission maximum) of 20–40 nm and broad emission lines. A sequential capturing of fluorescence images allows a higher number of fluorophores, but then the imaging of all single channels takes longer [19]. To extend the number of signals that can be recorded from one sample, dyes with higher Stokes shift are required. Such dyes are also of interest in super-resolution optical microscopy [20]. Besides the available organic dyes, there are many approaches with fluorescent nanoparticles [20–22] like quantum dots [23, 24] or ultrasmall gold nanoparticles/clusters [25], and with fluorescent proteins [26, 27].

Here, we chose ultrasmall gold nanoparticles as label for PLGA nanoparticles. These nanoparticles show a bright emission when excited [28]. The emission wavelength depends on various factors like the particle size [29], the valence state [30], the nature of the surface ligands [31], and the particle crystallinity [32, 33]. Unlike bigger gold nanoparticles, the luminescence mechanism of these ultrasmall particles cannot be described as plasmon resonance, but rather as ligand-to-metal or metal-to-ligand charge transfer [33]. The emission spectra of these nanoparticles are tunable from the IR to the UV range [34]. In contrast to some organic dyes, no cytotoxic degradation products are caused by photobleaching [27]. In general, such nanoparticles have a high photostability and, compared to organic fluorescent dyes, show only little photobleaching [35, 36]. Thus, ultrasmall autofluorescent gold nanoparticles are a promising fluorescent label for polymeric nanoparticle. Here we present a procedure to incorporate ultrasmall gold and gold/silver nanoparticles into PLGA nanoparticles that can act as carrier for drugs or biomolecules into cells [37].

## Experimental section

### Chemicals

A solution of tetrachloroauric acid (HAuCl_4_) was prepared by dissolving elemental gold (≥99%) in *aqua regia*. Sodium borohydride (NaBH_4_, ≥96%), 11-mercaptoundecanoic acid (95%), poly(D,L-lactide-*co*-glycolide) 50:50 (Resomer^®^ RG 502 H, *M*_w_ = 7000–17,000 g mol^−1^), polyvinyl alcohol (PVA, *M*_w_ = 30,000–70,000 g mol^−1^, 87–90% hydrolysed), polyethylenimine (PEI, branched, *M*w = 25 kDa), and ammonia solution (25%) were obtained from Sigma-Aldrich (St. Louis, MO, USA). Silver nitrate (≥99%) was obtained from Carl Roth (Karlsruhe, Germany). Dichloromethane was obtained in analytical grade (99.99%) from Fisher Scientific (Loughborough, UK). Degassed ultrapure water (Purelab ultra instrument, ELGA, Germany) was used for all syntheses and purifications unless otherwise noted. Prior to use, all glassware was cleaned with boiling *aqua regia* and thoroughly washed with ultrapure water afterwards.

### Synthesis of ultrasmall gold and silver/gold nanoparticles

Ultrasmall autofluorescent gold nanoparticles (AuNP) and alloyed silver/gold nanoparticles (AgAuNP) with a silver:gold ratio of 1:1 were prepared as described earlier [25, 38] by mixing 50 mL of either 500 µL 10 mM HAuCl_4_ (AuNP) or 250 µL 10 mM HAuCl_4_/250 µL 10 mM AgNO_3_ solution (AgAuNP). The mixture was stirred for 5 min, followed by adding 1 mL 11-mercaptoundecanoic acid (MUA) in ethanol (25 mM) and 1 mL of NaOH (50 mM). After stirring for 5 min, 200 μL of a solution of sodium borohydride (200 mM; 0 °C) was added dropwise and further stirred for 10 min. The solution was acidified by dropwise addition of 1 M HCl, precipitating the particles which were then purified by centrifugation (2900 g, 15 min). To remove unbound MUA, the precipitate was washed with 50 mL ethanol and then twice with water. Finally, the particles were redispersed in 5 mL of 0.1 M NH_3_. To determine the metal concentrations after purification of the nanoparticles, the particles were chemically dissolved, and the concentrations measured by AAS. To determine the Au concentration for pure AuNP, the particles were dissolved in *aqua regia*, whereas alloyed AgAuNP were dissolved in nitric acid. The intended molar of silver to gold ratio for alloyed AgAu nanoparticles was 1:1. Atomic absorption spectroscopy after dissolution showed that the metals were present in the ratio Ag:Au = 6:4.

### Synthesis of nanoparticle-labelled PLGA particles

The PLGA particles were prepared by a water-in-oil-in-water emulsion-evaporation-technique as described earlier [13, 37, 39]. Briefly, 10 mg PLGA were dissolved in 750 µL ice-cold dichloromethane. 250 µL of either the metal nanoparticle dispersion (AuNP: 3.7 g L^−1^; AgAuNP: 1.1 g L^−1^Au/1.2 g L^−1^ Ag) or of 0.1 M NH_3_ were added. The two phases were ultrasonicated for 20 s (amplitude 80%, pulse 0.8; Hielscher UP50H sonotrode, MS3). This water-in-oil-emulsion (W_1_/O) was rapidly added to 3 mL of a 1% solution of polyvinyl alcohol (PVA) and ultrasonicated for 40 s under the same conditions as before, forming a water-in-oil-in-water emulsion (W_1_/O/W_2_). Dichloromethane was removed by evaporation under stirring at room temperature for 12 h. To remove excess PVA, the dispersion was centrifuged (21,100 *g*, 15 min). The PLGA particles were redispersed in 1 mL water by ultrasonication for 10 s. This step was repeated three times for purification.

A positive surface charge for better cellular uptake was realised by adding an outer layer of polyethylenimine (PEI). The PLGA particles (10 mg) were dispersed in 1 mL water and added dropwise to 1 mL of a 2 g L^−1^ solution of PEI. The mixture was stirred for 30 min and the particles were purified three times by centrifugation and redispersion (21,100 g, 15 min). For additional fluorescent labelling, PEI-FITC (Surflay, Berlin, Germany) was used in a *w*:*w* ratio for PEI:PEI-FITC of 10:1.

In the following, the PLGA particles containing ultrasmall gold nanoparticles are denoted as PLGA/Au, the PLGA particles containing ultrasmall silver/gold nanoparticles are denoted as PLGA/AgAu, and the PLGA particles without metallic nanoparticles are denoted as PLGA/empty.

To determine the metal concentration in the nanoparticle-loaded PLGA particles, *aqua regia* was added to the particles and the dispersion was boiled for 10 min. The resulting solutions were analysed by AAS.

### Characterisation

Gold and silver were determined by atomic absorption spectroscopy (AAS) with a Thermo Electron M-Series spectrometer (graphite tube furnace according to DIN EN ISO/IEC 17025:2005). Analytical disc centrifugation (differential centrifugal sedimentation; DCS) was performed with a CPS Instruments DC 24000UHR disc centrifuge (24,000 rpm). Two sucrose solutions (8 and 24 wt%) formed a density gradient which was capped with 0.5 mL dodecane as stabilising agent. The calibration standard was a poly(vinyl chloride) latex in water with a particle size of 483 nm provided by CPS Instruments. The calibration was carried out prior to each run. A sample volume of 100 μL was used. Dynamic light scattering (DLS) for particle size analysis and zeta-potential determination was carried out on a Malvern Zetasizer Nano ZS ZEN 3600 instrument (25 °C, laser wavelength 633 nm). The scattering was monitored at a fixed angle of 173° in backward scattering mode. The primary data were derived from the correlation function of the scattered intensity as number-weighed size distribution.

Ultraviolet–visible spectroscopy (UV–Vis) and fluorescence spectroscopy were performed with an Agilent Cary Eclipse spectrophotometer from 200 to 600 nm (UV–Vis) and 550–800 nm (fluorescence) with background correction. Suprasil^®^ cuvettes with a sample volume of 3 mL were used. Scanning electron microscopy (SEM) was performed with an ESEM Quanta 400 instrument (FEI, Eindhoven) with gold/palladium-sputtered samples.

The cells were studied by confocal laser scanning microscopy (CLSM) with a Leica TCS SP8 with a 63×/NA 1.2 water objective with the cells seeded in an ibidi^®^ µ-Slide (Planegg, Germany). For the MTT essay, the spectrophotometric analysis was carried out in a 96-well plate with a Multiscan FC instrument (Thermo Fisher scientific, Vantaa, Finland) at 570 nm.

### Cell culture studies

Cell culture studies were carried out with human cervix carcinoma cells (HeLa). The cells were cultured in Dulbecco’s modified Eagle’s medium (DMEM) supplemented with 10% foetal bovine serum (FBS), 100 U mL^−1^ penicillin, and 100 U mL^−1^ streptomycin at 37 °C in a humidified atmosphere with 5% CO_2_.

The cytotoxicity (expressed as cell viability) was determined with a 3-(4,5-dimethylthiazol-2-yl)-2,5-diphenyltetrazolium bromide (MTT) cytotoxicity assay. The cells were trypsinized and seeded in a 24-well culture dish with 2.5 × 10^4^ cells per well in 500 µL cell culture medium 24 h prior to the experiments. For the incubation of cells with nanoparticles, 100 µL of a 2.5 g L^−1^ dispersion in DMEM was added to the medium. The cells were incubated for either 3 or 24 h. Afterwards the cells were washed three times with 500 µL PBS and incubated with 300 µL MTT solution (1 g L^−1^) for 1 h at 37 °C. Then the MTT solution was replaced by 300 µL DMSO and the incubation was continued for 30 min. Finally, sample triplicates were transferred into a 96-well plate (100 µL aliquots) for spectrophotometric analysis. The relative cell viability was calculated by comparison to a control group of untreated cells.

For nanoparticle uptake studies, the cells were cultured in DMEM supplemented with 10% FBS, 100 U mL^−1^ penicillin, and 100 U mL^−1^ streptomycin at 37 °C in a humidified atmosphere with 5% CO_2_. The cells were trypsinized and seeded in a glass bottom dish with 10^4^ cells per well in 150 µL cell culture medium 24 h prior to the addition of nanoparticles. After adding 40 µL of a 2.5 g L^−1^ PLGA-dispersion in DMEM to the cell medium, the cells were incubated for 24 h. Then the cells were washed three times with 150 µL PBS and fixed with 100 µL 4% aqueous paraformaldehyde for 20 min at room temperature and washed again three times with 150 µL PBS. Prior to actin staining, the cells were permeabilized with 150 µL 0.1% Triton X-100 for 5 min and washed twice with 200 µL PBS. For actin staining, the cells were incubated with 150 µL of 25 µg mL^−1^ Alexa Fluor^®^ 647-phalloidin (Invitrogen, Karlsruhe, Germany) solution in PBS with 1% bovine serum albumin (BSA). Afterwards the cells were washed three times with 150 µL PBS.

## Results and discussion

Ultrasmall gold nanoparticles (diameter 1–3 nm) which are meeting the size range of atomically sharp gold clusters [40–43] often show a high fluorescence in the visible part of the spectrum that has been well documented [33, 44–46]. To stabilise these ultrasmall nanoparticles, thiols, dendrimers or thiol-based polymers can be used [28, 47–51]. Particles stabilised with small molecules carrying thiol groups show fluorescence with low quantum yield (<1%) [47]. Negishi et al. prepared a series of glutathione-protected gold clusters with different atom numbers, giving emission maxima between 1.6 and 1.8 eV and Stokes shifts of 0.4–0.1 eV, decreasing with increasing number of gold atoms [52]. When stabilised by dendrimers, the quantum yield of the gold nanoparticles increases. Zheng et al. prepared gold clusters encapsulated in poly(amidoamine) dendrimers with 5–31 gold atoms, emitting from UV to IR range, with the emission wavelength increasing with the number of atoms in the cluster. The Stokes shift of these systems was 0.2–0.5 eV, with quantum yields of 10–70%, both decreasing with an increasing number of atoms. They found that the emission energy equals *E*_Fermi_/*N*^1/3^ with *E*_Fermi_ the Fermi energy of bulk gold, according to the jellium model [29]. Xie et al. prepared gold nanoclusters by reducing gold with BSA under basic conditions. The resulting particles were entrapped inside the protein. When excited at 480 nm, the particles emitted at 640 nm, giving a Stokes shift of 160 nm and a quantum yield of 6% [53]. Liu et al. prepared gold nanoparticles with a size of 2.5 nm by reducing the gold in the presence of glutathione with different ratios of gold to ligand and found different emission maxima for the resulting nanoparticles [54]. However, it must be stressed not all ultrasmall nanoparticles of noble metals show autofluorescence [55–57]. Gold nanoparticles and clusters are especially useful for this purpose.

The reduction of tetrachloroauric acid with sodium borohydride in the presence of 11-mercaptoundecanoic acid leads to ultrasmall gold nanoparticles [25, 38]. The lattice parameters of gold and silver are similar, allowing to prepare alloys in every ratio [57, 58]. Ristig et al. prepared alloyed ultrasmall silver/gold nanoparticles to vary the emission of the ultrasmall nanoparticles [25]. To obtain alloyed silver/gold nanoparticles, tetrachloroauric acid and silver nitrate were mixed in the desired silver to gold ratio prior to the reduction to obtain all combinations from 0:100–100:0 mol% Ag:Au in steps of 10% [25]. The particles were analysed by AAS with respect to their composition. The results showed a high controllability of the combination of the two metals with deviations of about ±3% to the intended value. The particle diameter (by differential centrifugal sedimentation, DCS) increased with increasing amount of Ag from 1.5 nm (100% Au) to 2.2 nm (100% Ag). These ultrasmall nanoparticles could be excited in the UV-range at 250 nm and emitted at about 620 nm [25]. The emission showed a red shift with increasing concentration of Ag, while alloys with more than 60% Ag did not emit anymore. Thus, to obtain autofluorescent nanoparticles, the concentration of 60% Ag should not be exceeded. We have prepared such ultrasmall silver/gold nanoparticles for fluorescent labelling.

**Fig. 1:**
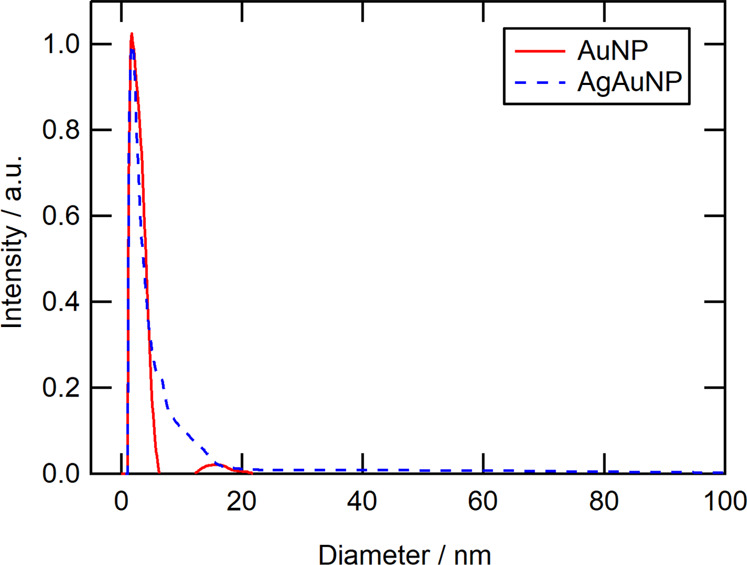
Disc centrifugal sedimentation analysis of ultrasmall gold nanoparticles (AuNP) and alloyed silver/gold nanoparticles (AgAuNP)

We prepared unlabelled PLGA nanoparticles (PLGA/empty), gold-labelled PLGA nanoparticles (PLGA/Au), and silver/gold-labelled PLGA nanoparticles (PLGA/AgAu). The size of both kinds of metallic nanoparticles was determined by differential centrifugal sedimentation (DCS), giving average diameters of 1.8 nm (AuNP) and 1.9 nm (AgAuNP) (Fig. 1). Note that the ligand shell has a lower density than the metallic core, therefore the actual size of the particles is systematically underestimated by DCS for ultrasmall nanoparticles [59].

**Fig. 2:**
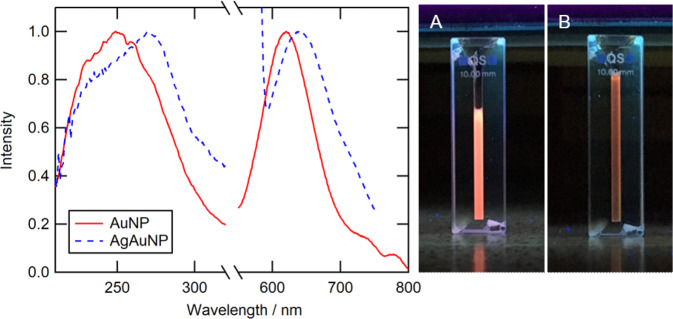
Left: Absorption (200–320 nm) and emission (550–800 nm) spectra of ultrasmall gold (AuNP) and silver/gold nanoparticles (AgAuNP) with a Stokes shift of 370 nm. Right: Fluorescence of ultrasmall nanoparticles under UV irradiation (254 nm): AuNP (**A**) and AgAuNP (**B**)

By excitation of dried as well as of dispersed particles with UV light, a bright orange emission is visible. UV–Vis spectroscopy shows an absorption maximum at 250 nm for AuNP and at 270 nm for alloyed AgAuNP. An excitation at 250 nm led to a fluorescence emission with a high Stokes shift with emission maxima at 620 nm for AuNP and 640 nm for AgAuNP (Stokes shift 370 nm in both cases) (Fig. 2).

**Fig. 3:**
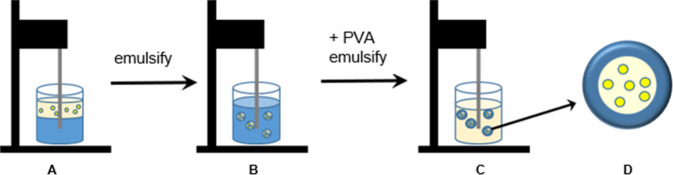
Schematic representation of the PLGA particle synthesis by a water-in-oil-in-water emulsion-evaporation method. **A** Dispersion of ultrasmall gold nanoparticles in water and dissolution of PLGA in dichloromethane; **B** Water-in-oil dispersion; **C** Water-in-oil-in-water dispersion, stabilised by PVA; **D** Schematic image of a PLGA particle, containing ultrasmall gold nanoparticles

Figure 3 shows the synthesis of the PLGA nanoparticles labelled with ultrasmall metallic nanoparticles incorporated as fluorescent labels.

**Fig. 4:**
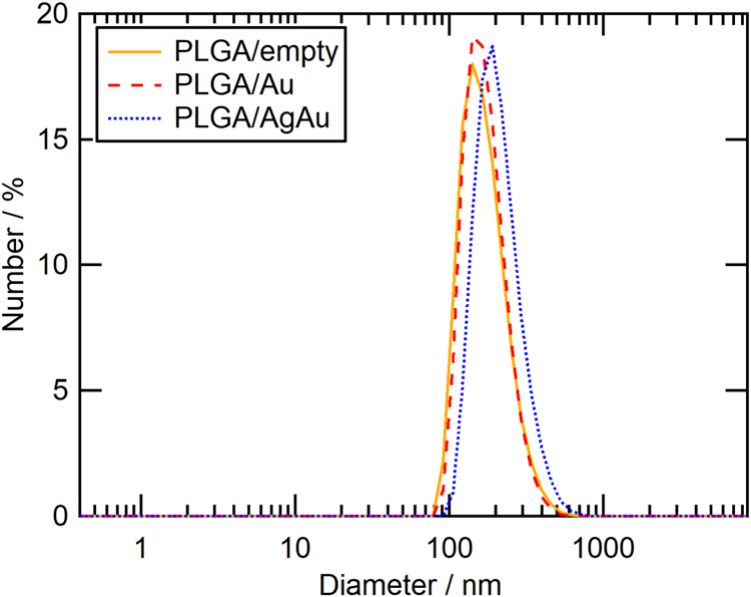
Dynamic light scattering data of PLGA nanoparticles without (PLGA/empty) and with ultrasmall metal nanoparticles (PLGA/Au and PLGA/AgAu)

The hydrodynamic diameter and the zeta-potential of the PLGA nanoparticles were determined by DLS. Empty and loaded PLGA nanoparticles showed hydrodynamic diameters in the same size range (225–230 nm) with low polydispersity indices, i.e. the presence of the ultrasmall nanoparticles did not influence the PLGA particle synthesis (Fig. 4). The zeta-potential was highly positive, indicating a high electrostatic colloidal stability of the nanoparticles due to the presence of the outer layer of the cationic polyelectrolyte PEI. The analytical results for all nanoparticles are summarised in Table 1.

**Table 1:** Results of dynamic light scattering and zeta-potential measurements of the different PLGA nanoparticles

Sample	Particle diameter by DLS/nm	PDI by DLS	ζ-Potential by DLS/mV	Particle diameter by SEM/nm
PLGA/empty	231 ± 8	0.13 ± 0.04	+28 ± 12	145 ± 30
PLGA/Au	225 ± 36	0.21 ± 0.04	+29 ± 8	128 ± 31
PLGA/AgAu	229 ± 49	0.18 ± 0.03	+36 ± 7	128 ± 32

The average and the standard error were calculated from synthesis triplicate.

**Fig. 5:**
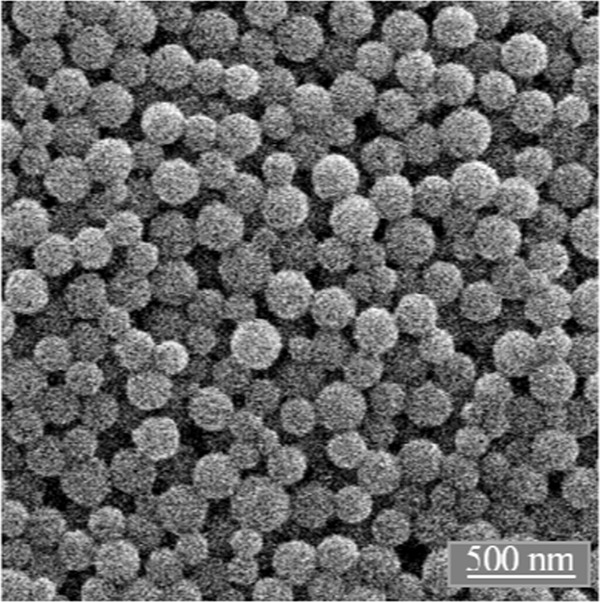
Scanning electron micrograph of PLGA/empty nanoparticles. PLGA/Au and PLGA/AgAu nanoparticles looked very similar

SEM showed spherical nanoparticles with good monodispersity and a diameter of 130–150 nm (Fig. 5). The fact that the diameter by DLS was only about 50% larger than the diameter by SEM indicates that the particles were well dispersed in water.

**Fig. 6:**
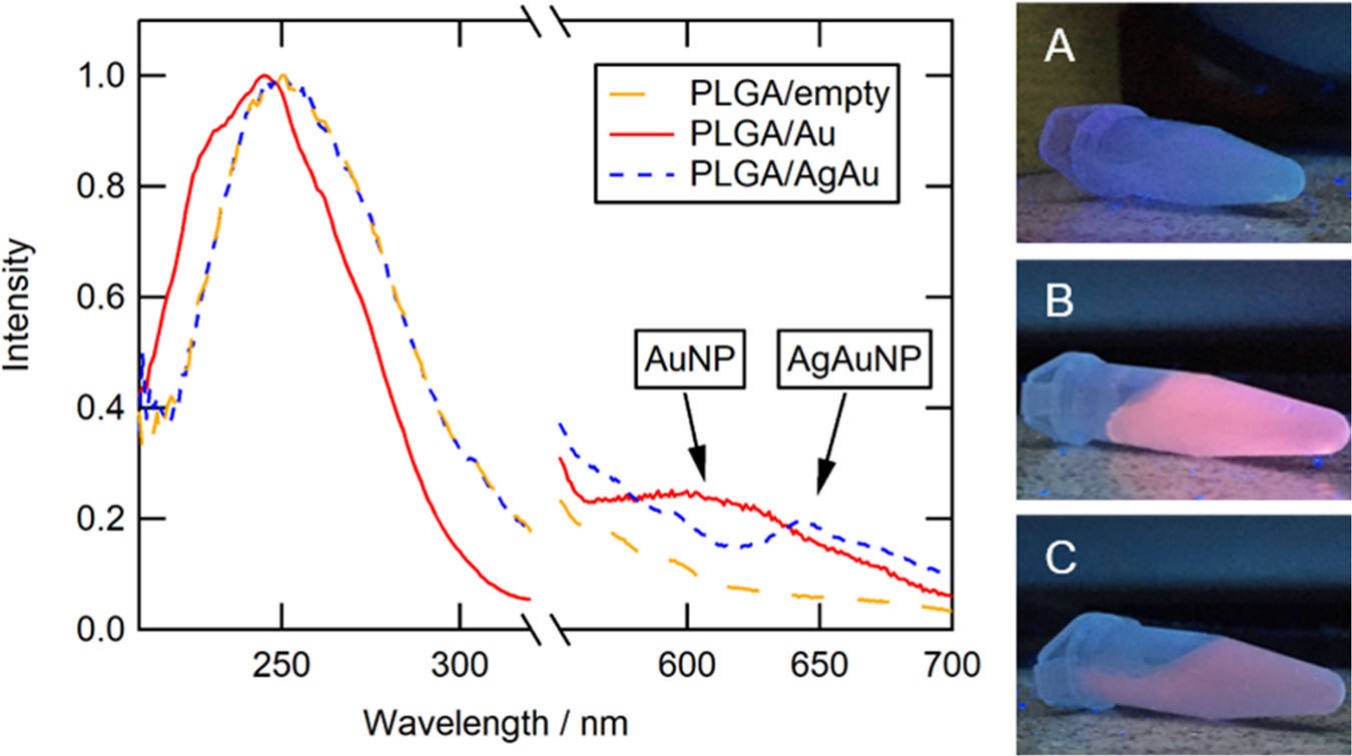
Left: Absorption (200–320 nm) and emission (550–800 nm) spectra of PLGA/Au, PLGA/AgAu, and PLGA/empty nanoparticles. Right: Images of PLGA nanoparticles under UV light irradiation (254 nm): PLGA/empty (**A**), PLGA/Au (**B**), and PLGA/AgAu (**C**)

Figure 6 shows UV–Vis and fluorescence spectra of the PLGA nanoparticles. The absorption maxima were 245–250 nm for PLGA/empty, PLGA/Au, and PLGA/AgAu nanoparticles. Clearly, the absorption band of the metallic nanoparticles overlaps with that of PLGA. Upon excitation at 250 nm, the emission maxima of the metallic nanoparticles are 605 nm for PLGA/Au and 645 nm for PLGA/AgAu. Compared to the pure metallic nanoparticles, the emission bands became broader after the encapsulation. The total fluorescence intensity was lower due to the dilution in the polymer matrix. As expected, the empty nanoparticles did not show fluorescence.

**Fig. 7:**
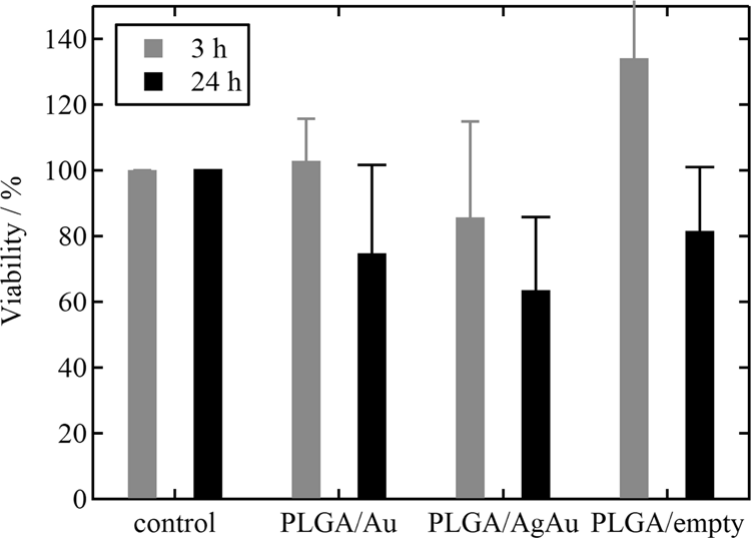
Viability of HeLa cells as determined by the MTT test after incubation with PLGA nanoparticles for 3 h (grey) and 24 h (black). The data points represent the mean value with standard error of sample triplicates. Untreated cells were used as control (viability 100%). There was no statistically significant difference between the groups

The cytotoxicity of such labelled PLGA nanoparticles and their uptake by cells were studied. The cell viability was measured by the MTT essay after 3 and 24 h incubation time with nanoparticles, in comparison to untreated cells (Fig. 7). At both times, there was no significant cytotoxicity of the nanoparticles in any group.

**Fig. 8:**
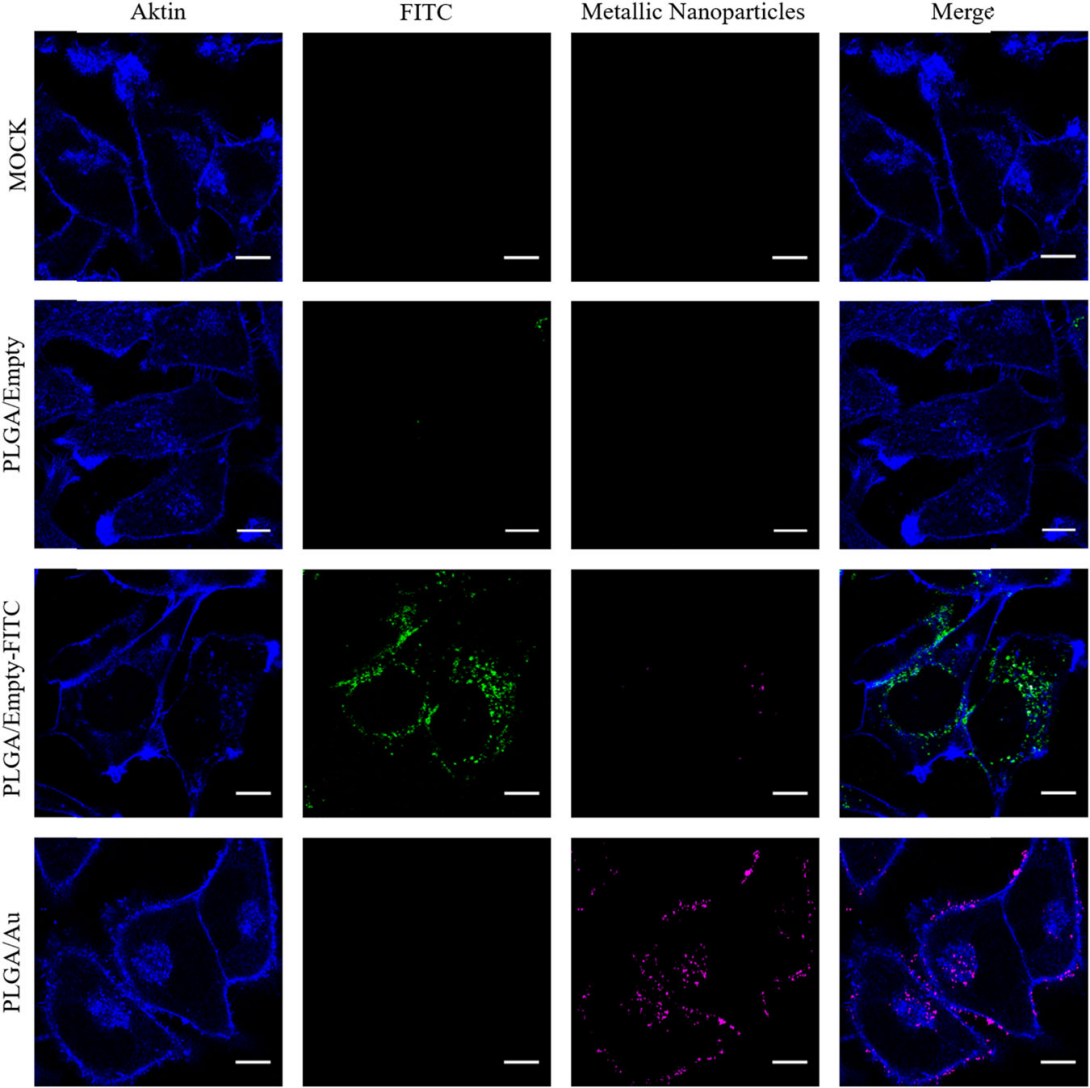
Confocal laser scanning microscopic images of HeLa cells after incubation for 24 h with PLGA nanoparticles in single channels and merge: Untreated cells (mock); PLGA nanoparticles without metallic nanoparticles but labelled with FITC (PLGA/empty-FITC); PLGA nanoparticles labelled with ultrasmall gold nanoparticles (PLGA/Au). Nanoparticles labelled with ultrasmall silver/gold nanoparticles (PLGA/AgAu) showed very similar results. Blue: Actin staining of the cytoskeleton; green: FITC-label of the PEI-FITC nanoparticles; magenta: Autofluorescence of the ultrasmall nanoparticles (Au). Scale bar: 10 µm

The uptake of PLGA nanoparticles by HeLa cells was studied after an incubation for 24 h by confocal laser scanning microscopy (CLSM). For this, the PLGA/empty nanoparticles were additionally labelled with PEI-FITC to make them visible in the CLSM. Figure 8 shows the details of the nanoparticle uptake. All three kinds of particles were well taken up by the cells. The fluorescence of the metallic nanoparticles was comparable to the FITC signal, demonstrating their suitability for fluorescent labelling.

**Fig. 9:**
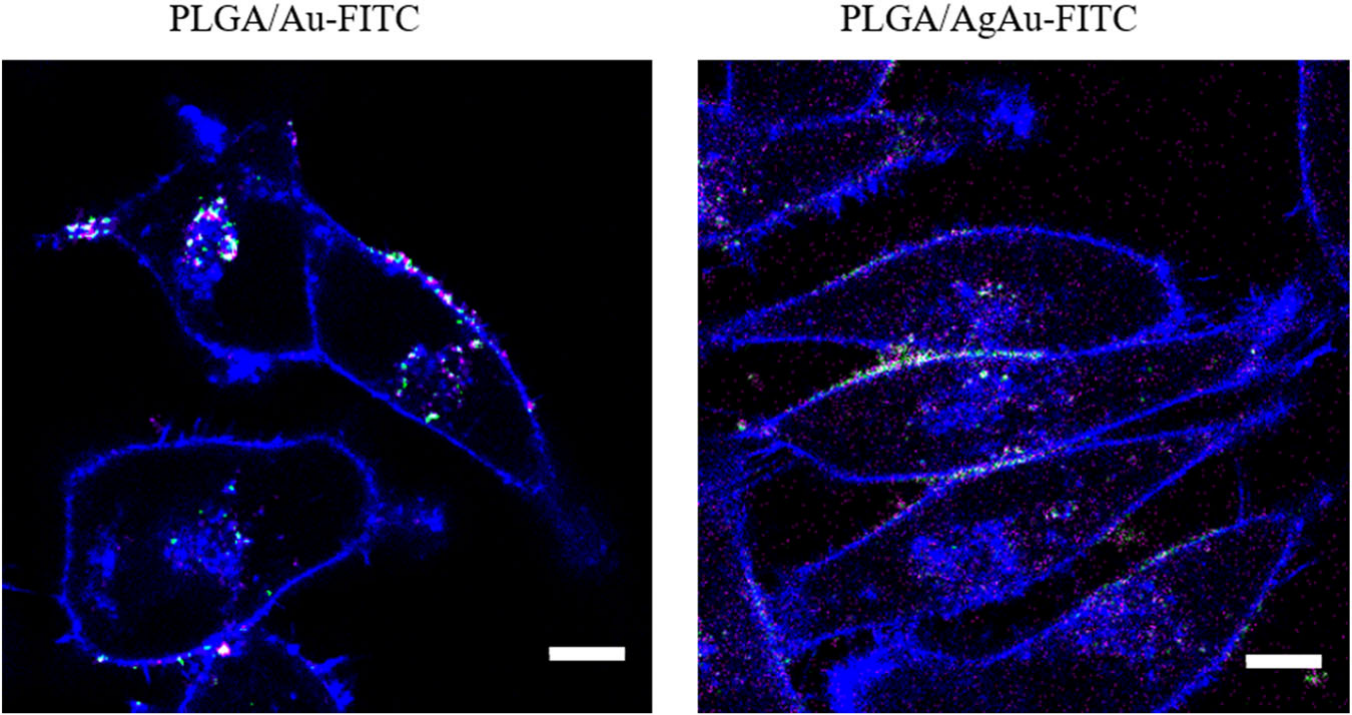
Confocal laser scanning microscopic images of HeLa cells after incubation for 24 h with PLGA/Au-FITC (left) and PLGA/AgAu-FITC (right). The cytoskeleton is shown in blue, the metallic nanoparticles in magenta and the FITC label of the nanoparticles in green. White areas indicate the colocalization of metallic nanoparticles and of FITC-labelled nanoparticles. Almost all particles appear in white or at least magenta and green in close vicinity. Scale bar: 10 µm

Although the ultrasmall metallic nanoparticles have their absorption maximum at 250 nm which is well below the lowest excitation wavelength of the confocal microscope (405 nm), the emission of the ultrasmall nanoparticles was strong. An excitation at a lower wavelength would clearly enhance the emission. To further examine the colocalization of metallic nanoparticles and PLGA-shell, FITC-labelled PLGA nanoparticles loaded either with AuNP or AgAuNP were given to the cells (PLGA/Au-FITC and PLGA/AgAu-FITC). Almost all particles appeared in white (=colocalization) or at least magenta (metallic nanoparticles) and green (=FITC) in close vicinity. The high degree of colocalization (Fig. 9) shows that the metallic particles remained encapsulated within the PLGA particles after the uptake. As the uptake will eventually lead into an endolysosome [60–64], the metallic nanoparticles will finally be released from the PLGA matrix after its hydrolytic degradation.

## Conclusions

Ultrasmall gold and alloyed gold-silver nanoparticles (2 nm) show autofluorescence with high Stokes shifts (about 370 nm). By varying the ratio of Ag:Au, the absorption and emission wavelengths can be tuned. Thereby, variable fluorescence labels can be obtained that can be custom-made for the desired emission for bioimaging. Ultrasmall gold and silver/gold nanoparticles (diameter 2 nm) were successfully encapsulated into PLGA nanoparticles (diameter about 140 nm) by a water-in-oil-in-water emulsion method. Due to their small size, the ultrasmall nanoparticles can serve as fluorescent labels for polymer nanoparticles, e.g. for uptake and intracellular distribution studies with cells. The particles remained incorporated in the polymer particles after uptake by cells. An excitation in the UV range at about 250 nm would take the full advantage of the high Stokes shift of these nanoparticulate labels for intracellular imaging and tracing polymer nanoparticles inside cells. An additional loading of the PLGA nanoparticles with drugs or biomolecules is easily possible by the presented emulsion synthesis.
